# Current Perspectives and Challenges of MAIT Cell-Directed Therapy for Tuberculosis Infection

**DOI:** 10.3390/pathogens12111343

**Published:** 2023-11-12

**Authors:** Melissa D. Chengalroyen

**Affiliations:** Molecular Mycobacteriology Research Unit, Institute of Infectious Disease and Molecular Medicine, Department of Pathology, University of Cape Town, Cape Town 7700, South Africa; mel.chengalroyen@uct.ac.za

**Keywords:** MAIT cells, tuberculosis, MR1-dependent, MR1-independent

## Abstract

Mucosal-associated invariant T (MAIT) cells are a distinct population of non-conventional T cells that have been preserved through evolution and possess properties of both innate and adaptive immune cells. They are activated through the recognition of antigens presented by non-polymorphic MR1 proteins or, alternately, can be stimulated by specific cytokines. These cells are multifaceted and exert robust antimicrobial activity against bacterial and viral infections, direct the immune response through the modulation of other immune cells, and exhibit a specialized tissue homeostasis and repair function. These distinct characteristics have instigated interest in MAIT cell biology for immunotherapy and vaccine development. This review describes the current understanding of MAIT cell activation, their role in infections and diseases with an emphasis on tuberculosis (TB) infection, and perspectives on the future use of MAIT cells in immune-mediated therapy.

## 1. Introduction

Immunity is reliant on two levels of defense; innate and adaptive immunity. The innate response, classically labeled as the ‘first line of defense’, relies on the broad recognition of microbial proteins or sugars, which upon activation, induces phagocytosis or apoptosis, cytokine release, and degranulation [1]. This generalized response can either eliminate or prevent further proliferation of the pathogen; the failure of which allows proliferation of the pathogen and subsequently provides epitopes capable of activating the adaptive response. T cells are a central part of orchestrating adaptive immunity and act by scanning the surface of antigen-presenting cells for major histocompatibility complex (MHC) molecules that present foreign polymorphic antigens, commonly in the form of antigenic peptides. These cells undergo clonal expansion in the thymus and differentiate into effector and memory T cells, allowing an immune memory against the antigen to be established. Conventional T cells play an important role in either killing infected host immune cells or activating other immune cells to regulate host immunity against a disease or to control an infection. A subset of T cells, classified as unconventional T cells, which includes invariant natural killer T (iNKT) cells, gamma-delta (γδ) T cells, and mucosal-associated invariant T (MAIT) cells, is characterized by their ability to recognize antigens presented by unconventional antigen presenting molecules and to elicit cytokine responses in a T cell receptor (TCR) independent manner. The γδ-T cells (characterized by TCRs, TRGV9-TRDV2 [Vγ9Vδ2]) recognize lipid antigens presented by CD1 molecules [2], while human iNKT cells (characterized by TCRs, Vα24-Jα18, and Vβ11) [3] recognize phospho-antigens, which are intermediates of the isoprenoid biosynthesis pathway, presented by the CD1d molecule [4]. MAIT cells are discussed in more detail below.

## 2. MAIT Cell Development and the Microbiota

MAIT cells bridge innate and adaptive immunity [5,6]; like innate immune cells, they are the first line of defense, capable of mounting a robust immune response in an antigen-dependent/independent manner, serving as rapid responders to invasive bacterial infections, and like adaptive immune cells, attain effector memory. Single-cell RNA-sequencing comparing gene expression normally associated with innate versus adaptive immunity found that MAIT (as well as iNKT and Vδ2 T) cells expressed transcriptional profiles associated with both states of immunity, although they were transcriptionally closer to conventional T cells than to innate cells [7]. MAIT cells express the transcriptional regulators promyelocytic leukemia zinc finger (PLZF), RORγt, and T-bet, all vital for different stages of development [8,9]. These cells undergo three stages of development; initially defined as CD27−CD161− at stage 1, transitioning to CD27+CD161− in stage 2, and CD27lo/+CD161+ at stage 3 [10]. They develop in the thymus as naive cells and are postulated to be dependent on exposure to bacterial antigens of commensal microflora for further development and maturation in the periphery [10,11]. The antigens traffic from the mucosa to the thymus, which enhances MAIT cell development and expansion [12]. However, recent research has also identified MAIT cells in second trimester human fetal tissue prior to the establishment of gut microbiota [13]. Work carried out in a murine model showed that MAIT cell development occurs within a short period in the early stages of life and is strongly influenced by intestinal microbiota. This initial microbial exposure is vital in that it also determines MAIT cell abundance for life [14]. These cells range from 1–10% in blood, 2–10% in the intestine, and 20–50% in the liver [15], altogether comprising around 25% of CD8 T cells. These cells are distinguished by the markers CD161; receptors for IL-7, -12, -18, and -23; CD26; and C-C chemokine receptor 6 (CCR6), CXCR6, and CCR5. The TCR α-chain consists of TRAV1-2 with either TRAJ33/12/20 paired with variable β-chains in humans, generally TRBV6-1, TRVB 6-4 or TRBV20 [16]. The α-chain is highly evolutionarily conserved amongst mammals [17]; hence, conformational changes within the complementarity-determining region (CDR) 3β loop directs affinity for the MR1-presenting antigen [18].

A comprehensive analysis of 50 types of immune cell populations in human peripheral blood from birth to old age (75 years) showed that MAIT cells were low in the first years of life (<0.08%), increased in young children (~2.3%), peaked in adults aged 19–30 years (4.3%), and lowered again in old age (~0.9%) [19]. The same study found that CD57+ NK cells, and CD4+ and CD8+ memory T cells increased in late adulthood while dendritic cells declined. A deeper understanding of this natural shift in immune status throughout life could guide therapies to boost unconventional and conventional immune cells to protect vulnerable populations against infections. 

## 3. Features of MAIT Cells

**Figure 1:**
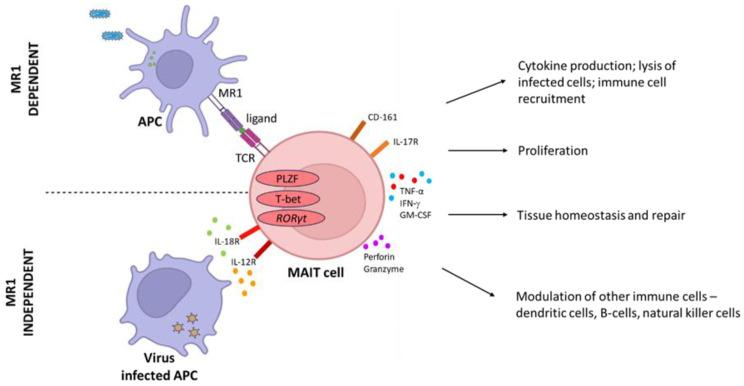
MAIT cell activation via MR1-dependent and MR1-independent pathways. These cells have been shown to play notable roles in response to bacterial and viral infections, autoimmune conditions, vaccine-induced immunity, tissue repair, and tissue homeostasis. Created with BioRender.com.

The main distinguishing feature of MAIT cells is their ability to recognize monomorphic MHC-1-related molecules (MR1), which present precursors from the riboflavin (RF) pathway present in many bacterial species, making them rapid responders to bacterial infection. MAIT cell-dependent activation by MR1 leads to the production of inflammatory cytokines IFN-γ, TNF-α, and IL-17 to control infection (Figure 1). In addition granzyme B and perforin are released to lyse-infected cells. The cytokines IL-12 and IL-18 [20], common signals from viral infection, activate MAIT cells in an MR1-independent manner [21], associating an innate-like activation mode to MAIT cells. In mice, MAIT cells are categorized into two subsets, MAIT17 cells, which produce IL-17 or MAIT1 cells, that produce IFNγ. Human MAIT cells, however, were found to be predominantly MAIT1 [22] with evidence that induction of the MAIT17 subset may occur during certain illnesses [23]. 

MAIT cells also modulate the activity of other immune cells to coordinate an effective adaptive immune response either through direct cell–cell contact or via signaling molecules. They play a role in the maturation of dendritic cells, the transactivation and cytotoxicity of natural killer cells [24], the reactivation and differentiation of memory B cells [25], the suppression of innate lymphoid cell proliferation [26], and the regulation of the Th1 response [27]. MAIT cell response is also mediated by costimulatory molecules of the immunoglobulin and TNF superfamilies, which is reviewed in detail by [28]. 

These cells are enriched in barrier tissues, locations that are most likely to come into first contact with pathogens and thus to incur a rapid inflammatory response that can cause tissue damage. A remarkable property of MAIT cells is that they express a tissue repair transcriptional profile [29,30]. Further studies showed that IL-22 and IL-17A released by the cells were linked to gut homeostasis [31]. Moreover, using a mouse skin injury model, it was demonstrated that MAIT cells produce amphiregulin, which aids in the closure of wounds, mediated in a CXCL16/CXCL6-dependent MR1-independent manner [32].

## 4. MR1-Bound Antigens

The earliest work in determining MAIT cell epitopes identified acetyl-6-formylpterin, a derivative of folate (vitamin B9), which although capable of binding MR1, did not activate MAIT cells [18,33]. The screening of small organic molecule and drug metabolite chemical libraries identified other molecules capable of interacting with MR1; a salicylic acid derivative, which is a by-product of the anticancer drug methotrexate, a portion of the immunosuppressant sirtinol, and the anti-inflammatory diclofenac, of which only diclofenac stimulated MAIT cell activity [34], establishing that certain drugs have the capacity to modulate MAIT cell activity. This has broader implications when considering immunotherapies to boost MAIT cell activity. 

By disrupting genes involved in RF biosynthesis in *Lactobacillus lactis*, 5-A-RU was identified as a central intermediate in MAIT cell modulation [35]. The most potent MAIT cell activators arise from 5-A-RU from the riboflavin (RF) pathway, which interacts with methylglyoxal/glyoxal derived from the glycolysis pathway to generate the unstable pyrimidines; 5-(2-oxoethylideneamino)-6-D-ribitylaminouracil (5-OE-RU) and 5-(2-oxopropylideneamino)-6-D-ribitylaminouracil (5-OP-RU) that stabilize upon MR1 binding [35]. It is peculiar that MR1 preferentially binds these transiently formed unstable intermediates and only weakly binds stable ligands from the RF pathway [36,37]. Other antigenic products include photolumazine-I (PLI) and -III (PLIII), again derived from 5-A-RU interacting with α-ketoglutarate [38]. The product 6,7-dimethyl-8-(d-ribityl) lumazine (RL-6,7-diMe), a precursor of RF and derivatives thereof, reduced 6-(hydroxymethyl)-8-D-ribityllumazine (rRL-6-CH2OH) and reduced 6-(hydroxymethyl)-8-(1-D-ribityl) lumazine (RL-6-Me-7-OH) are also MAIT cell activators [33]. It was demonstrated that RF-based antigens have varying degrees of potency; 5-OP-RU being the most potent, followed by 5-OE-RU and RL-6,7-diMe [39]. Following mycobacterial infection, a prominent ligand, 7,8-didemethyl-8-hydroxy-5-deazariboflavin (FO) (postulated to be formed via the interaction of 5-A-RU with a by-product of the tyrosine pathway) was identified as a MAIT cell inhibitor [38]. Moreover, RF itself was also shown to be a weak MAIT cell antagonist [38]. It is interesting that 5-A-RU can yield products that are either activators or antagonists of MAIT cell activity, depending on the by-product with which it interacts [38]. The identification of MAIT cell inhibitors and activators emanating from the mycobacterial RF pathway suggests a complex mechanism of immunoregulation. Although further studies have not shown whether *Mtb* may exploit this phenomenon to mitigate the activation of MAIT cells. In line with this, it would be of interest to assess whether the metabolic state of *Mtb* influences the ratio of antagonist/activator, skewing the immune response. An intriguing finding established that microbe metabolism is, in fact, intricately linked to MAIT cell modulation. *E. coli* was grown under different conditions with altered carbon sources at varying concentrations and exposure to different pH and oxygen levels to resemble host physiological conditions. Ligands extracted from bacteria at a stationary growth stage and under anaerobic conditions most potently activated MAIT cells, correlating to an increase in activating ligands [39]. This study provides strong evidence that the host microenvironment that influences bacterial metabolism is an important factor in modulating MAIT cell behavior. 

Many studies have established that bacteria colonizing the human gut-microbiota produce MAIT cell ligands, giving rise to the question of why these cells are not constantly activated in the gut. It stands to reason that a symbiotic relationship must be maintained to prevent an inflammatory type response [40]. Colon biopsies showed that ~50% of MAIT cells expressed CD137+, a marker of activation. However, ~50% expressed the inhibitory receptors TIGIT+PD-1+, indicating that the cells are at different standby states. It was postulated that inhibitory molecules may be responsible for modulating this immune balance. Additionally, the activation of MAIT cells require both a TCR and cytokine signal (from pathogen recognition); hence, the absence of the secondary cytokine signals from gut microbes prevents activation.

## 5. MAIT Cell Response to Infection and Disease

Their distinctness in recognizing ligands from the microbial RF biosynthesis pathway indicates that a wide range of bacteria, namely *Helicobacter pylori*, *Salmonella typhimurium, Escherichia coli*, *Mycobacterium tuberculosis* (*Mtb*), *Shigella flexneria*, and *Staphylococcus aureus*, all with intact RF biosynthetic pathways, are recognized by these specialized cells [41,42,43]. The reader is referred to [44] for a comprehensive list of MAIT cell activating microbes. Notably, viruses (encephalomyocarditis, Sendai, Newcastle disease, Herpes simplex and Parainfluenza3 viruses) all fail to elicit a MR1-dependent MAIT cell response [45] because they do not generate these ligands. Since humans lack the ability to synthesize RF and accordingly cannot manufacture RF precursors, these antigens are highly specific for microbial invasion, making them important ligands for immunosurveillance. Interestingly, MAIT cell response towards *S. pneumoniae* clinical isolates differed based on differential expression of RF pathway genes. This difference is proposed to correspond to variation in the generation of RF-based antigens [46] and alludes to the possibility of downregulation of this pathway as a potential evasion strategy. There is also evidence of MAIT cell stimulation from pathogens that lack a RF biosynthetic pathway; in particular, recognition of the RF auxotroph *Streptococcus pyogenes* by MR1 [47], suggesting that alternate ligands other than those arising from the RF route may also act as activators. However, it is accepted that RF-related antigens appear to be the dominant stimulatory ligands. 

Bacterial infection is associated with a decrease in MAIT cells in blood and, in most documented cases, but not all, a concomitant increase at the site of infection [42,48,49]. Many studies reflect that susceptibility to bacterial infections increases in the absence of MAIT cells and is accompanied by enhanced pathology [49,50]. A reduction in circulating MAIT cell frequency is also observed in patients with cystic fibrosis [49], systemic lupus erythematosus [51], diabetes [52], sepsis [53], and cancer [54]. Depletion of circulating MAIT cells has also been observed in patients with severe bacterial sepsis and correlated with a higher incidence of intensive care unit acquired infections [53]. There is reported ambiguity regarding whether MAIT cells are protective in the inflammatory disease multiple sclerosis [55], indicating that the pro-inflammatory nature of these T cells is not definitive in this contextual state and emphasizes that a greater mechanistic understanding of the dysregulation of these cells is necessary in specific diseases. An early study showed that MAIT cells were not activated in response to several viruses [45]; however, this was carried out using Vα19-Vβ 6 transgenic T cells which do not express a transcription factor required for the expression of IL-12 and IL-18 [56]. Subsequent work has now shown that MAIT cells require IL-12 and IL-18 stimulation to respond to viral infections. 

In relation to non-bacterial infections, MAIT cell response is mediated in an MR1-independent manner through the choreographed release of distinct cytokines. For instance, viral infections (dengue, hepatitis C, and influenza viruses) trigger activation by IL-18 release in combination with IL-12 and IL-15 and/or IFN-α/β [57,58]. During early HIV infection, MAIT cells are significantly reduced in both peripheral blood and tissue and fail to recover following successful treatment with highly active antiretroviral therapy (HAART) [59], likely as a result of exhaustion and eventual depletion [60]. It is possible that the decline in MAIT cell populations may contribute to increased bacterial infections observed in HIV infected individuals. MAIT cells are also stimulated in an MR1-independent manner in inflammatory bowel diseases [61], lupus [51], type 2 diabetes [52], and certain cancers [54]. Obesity-linked type 2 diabetes associated with gut microbiota dysbiosis showed a clear correlation with decreased MAIT cell activating ligands by gut microbiota and increased inflammation mediated by MAIT cells [62]. MAIT cells in the adipose tissue of diabetics displayed a pro-inflammatory phenotype associated with elevated levels of IL-17 that improved following bariatric surgery [52]. Similarly, a higher frequency of MAIT cells producing pro-inflammatory IL-17 was also detected in children with asthma [63]. Since many autoimmune conditions are believed to be linked to dysbiosis of the gut microbiota [64] and MAIT cell activation is intricately linked to microbial RF pathway antigens, it is plausible to infer that MAIT cell activity may be dysregulated in these metabolic diseases. 

In pulmonary bacterial infections, MAIT cells have been shown to play a vital role in containment of disease, such as *Hemophilus influenzae,* through the release of granzyme B, carefully regulated by IL-12 and IL-7 signaling [58]. With respect to *Mtb* infection, MAIT cells are present in the airways and enriched in bronchoalveolar fluid of patients with pulmonary TB [65]. It has also been observed that MAIT cells are reduced in the blood of individuals with active TB and become enriched in the lung [41]. Additionally, these lung MAIT cells were highly reactive to *Mtb*-infected lung epithelia ex vivo. Although this signified that these cells are primary responders to early *Mtb* infection, further work was not conducted to indicate whether migration to the lungs was protective against infection. Of note, a polymorphism in MR1 in humans is strongly associated with susceptibility to TB infection [66].

## 6. MAIT Cells and Vaccine Development

There is only one licensed vaccine against TB, the Bacillus Calmette–Guérin (BCG) vaccine, which uses an attenuated strain of *Mycobacterium bovis*, which causes bovine TB. The BCG vaccine offers protection against different forms of TB in infants and children younger than 5 years, but only confers minor protection against extrapulmonary TB in children and is not correlative with protection against extrapulmonary TB in adults [67]. Overall, the effectiveness of BCG vaccination against all forms of TB is just 18% [67]. Furthermore, the vaccine is shown to indirectly offer protection against allergies, viral infections, and autoimmune ailments [68], known as an off-target effect, possibly by stimulating CD4+ and CD8+ memory T cells [69]. However, the fact that the efficacy of BCG vaccination wanes in adolescents and adults implies that alternative strategies to priming conventional T cells should be explored. To this extent, *M. bovis* BCG was genetically modified to overexpress a RF pathway gene to enhance the production of a proposed MAIT cell ligand and used to immunize mice. While there was a moderate reduction in bacillary load in the lungs and spleen compared to unimmunized mice, overall, no improved containment of the pathogen was observed [70]. 

From all of the evidence supporting MAIT cell expansion in response to both bacterial and viral infections, it is reasonable to conclude that microbe-based vaccines robustly activate the MAIT cell pathway. Yet it is only recently that there has been a shift in the focus from monitoring conventional T-cell responses to non-conventional T cells in vaccine studies. In this regard, controlled infection of healthy volunteers with live *Salmonella paratyphi* was used to track the standard response of MAIT cells to an infection. In this study, as observed in others, circulating MAIT cells decreased rapidly (within four days) and interestingly, cell frequency was restored following antibiotic treatment [71]. This is unlike what is observed during HIV infection, whereby MAIT cell numbers do not recover. An attenuated strain of *Shigella dysenteriae* tested as a vaccine candidate in humans led to the activation of MAIT cells, which effectively lysed infected cells and induced a B cell response [45]. MAIT cells that were challenged with *S. typhimurium* and adoptively transferred to immunodeficient mice were protective against lethal *Legionella* infection, while vaccination of mice by antigen priming prior to infection also enhanced protection [72]. Using a mouse model, the incorporation of the MR1 ligand, 5-OP-RU into vaccine formulations enhanced the pro-inflammatory response of MAIT cells against the influenza virus, effectively controlling the infection [73]. Moreover, the efficacy of this approach was maintained in young and old mice (keeping in mind that MAIT cell numbers naturally decline in old age) and this effect was replicated in human blood cultures. Immunization of mice with a *Salmonella* vaccine strain expanded MAIT cells into two distinct populations, one of which offered protection against bacterial *S. pneumonia* infection and the other which protected against the influenza virus [74]. Replication-incompetent adenovirus (Ad) vectors demonstrating strong cellular and humoral responses have been approved for use against the Ebola virus [75] and have shown potential in treating SARS-CoV-2 [76] and HIV infection [77]. Immunization with chimpanzee adenovirus Ox1 correlated with potent MAIT cell activation and concomitant upregulation of cytokine signaling pathways, augmenting CD8+ T cell immunity [78]. Clearly, from these studies, there is considerable evidence to support boosting MAIT cells as an effective strategy in long term protection against a variety of bacterial pathogens and viruses. 

## 7. MAIT Cell Directed Therapy and TB Infection 

Mice are not ideal models for studying MAIT cells since this population only makes up 0.05–0.6% of T cells compared to 1–50% within humans, depending on the tissue site [78,79]. However, models have been created to increase the abundance of MAIT cells by artificial boosting with the RF-based antigen, 5-A-RU in combination with methylglyoxal and the addition of a co-stimulus, leading to an 80-fold increase in liver MAIT cells and 40-fold increase in lung MAIT cells [80]. Also, genetically modified mouse models have been created, for instance, the Mr1+ Rorcgt-GFPTG B6-MAITCAST mouse, which produces 20 times more MAIT cells and is genetically modified to express specific human MAIT cell receptors [81]. Searching for a model organism more closely resembling humans has also led to the establishment of a macaque animal model for MAIT cell targeted vaccine development [82]. 

In light of studies showing the critical importance of MAIT cells in controlling bacterial pulmonary infections [48], it was surprising that *Mtb* mouse infection studies did not fully support a MAIT cell-dependent protective role. The priming of MAIT cell expansion with 5-OP-RU prior to *Mtb* infection failed to contain the infection [83], a finding supported independently in a similar study [84]. This was attributed to a delay in CD4 T cell priming dependent on TGF-β. However, administration of 5-OP-RU during chronic *Mtb* infection was shown to reduce the bacterial burden due to the induction of IL-17. The authors proposed that with antigen priming prior to infection, MAIT cells may have assumed a ‘tissue homeostasis’ state, whilst during *Mtb* chronic infection they adopt an antimicrobial phenotypic state, enhancing their ability to control the infection [83]. In contrast to this, in another murine model study, MAIT cells induced with 5-OP-RU and an agonist were unable to restrict chronic *Mtb* growth, yet effectively contained *M. bovis* BCG growth [85]. These studies focused on solely boosting MAIT cell activity, however, another potential approach might be to simultaneously stimulate other non-conventional T cell populations. Nonetheless, these murine model studies did not strongly support the notion of a protective effect of MAIT cells against *Mtb* disease, hence it was speculated that this may be as a result of significant differences between MAIT cells in mice and humans, in relation to both cell frequency and cell receptors [22,86].

In a rhesus macaque model monitoring active TB, latent TB, and coinfection with simian immunodeficiency virus (SIV), the MAIT cell population expanded and migrated to the site of infection, instigating a Th1 effector response [87]. Following 15 weeks of infection, described by the authors as a short-lived response, the level of MAIT cells returned to normal. It was difficult to interpret from these various disease state models whether MAIT cells contributed to controlling the infection, although it is clear that in a non-human primate model, MAIT cell response to the disease is rapid. A rather surprising outcome from another study showed that priming MAIT cells with 5-OP-RU in *Mtb*-infected rhesus macaques did not lead to expansion of the population, failed to stimulate cytokine production, and instead upregulated programmed death-1 (PD-1), normally associated with T cell exhaustion [88]. This dramatic reduction in circulating MAIT cell is also witnessed with HIV infection and HIV-TB coinfection and importantly, these numbers do not recover despite successful treatment, conceivably influencing the ability to withstand future opportunistic infections [59,89]. This suggests that MAIT cell replenishment could be a useful strategy to prevent disease relapse or other secondary infections. To this extent, work has been undertaken to reprogram induced pluriopotent stem cells to create MAIT cells, referred to as redifferentiated MAIT-like cells (reMAIT) [90]. The functionality of these T cells was demonstrated by infecting mice with *Mycobacterium abscessus* and introducing reMAIT, which exhibited effective host protection. This was driven by migration to various infected tissues with enhanced pathogen control (colony-forming unit reduction of 40–50%) via granulysin release [90]. Moreover, these cells matured and expanded in vivo, attaining extra T cell receptors. The priming of reMAIT cells by exposure to mycobacterial MAIT-activating antigens could potentially enhance the specificity of this strategy and mitigate the effect of T cell exhaustion.

However, this might not be equally effective against all pathogens; for instance, the Herpes simplex virus downregulates MR1 cell surface expression as a strategy to evade detection by MAIT cells [91]. Also, MAIT cells were demonstrated to be hyperresponsive to staphylococcal and streptococcal superantigen virulence factors, responsible for triggering a hyperinflammatory response (cytokine storm) quickly followed by cell exhaustion [92], a phenomenon that can be deleterious to the host and a clear indication of the risk of uncontrolled MAIT cell stimulation.

## 8. Concluding Remarks and Future Perspectives

When first discovered two decades ago, MAIT cells [17] were initially thought to serve solely as sentinels against bacterial infections. However, it has become apparent with further research that these specialized T cells have a more expansive role, responding additionally to a wide range of human diseases, viral infections, and the maintenance of tissue homeostasis.

MAIT cell response is driven by cytokine signals and influenced by interactions with other immune cells, with inherent host heterogeneity based on microbiota, host metabolism, diet, administered medication, past exposure to infection/s, and autoimmune ailments; an interplay of complex factors that is a challenge to immunotherapy. Whilst studies have demonstrated the potent antimicrobial effect of MAIT cells, the contribution of these cells to the immunopathology of specific diseases cannot be overlooked. Hence, whilst the rapid boosting of MAIT cells could be a beneficial strategy for the initial control of certain infections, protracted therapy may negatively impact other diseases within the host. In this regard, the integration of MAIT cell-directed therapy with personalized therapy, which incorporates the health and genetic profile of an individual, could be a means to overcome some complexities associated with immuno-therapeutics.

Initial studies against select pathogens tantalizingly revealed the critical importance of MAIT cells in responding to and effectively controlling both bacterial and viral infections. Paradoxically, findings stemming from the use of MR1 ligands as vaccine adjuvants in both murine and non-human primate models for use against *Mtb* infection have thus far not convincingly demonstrated a MAIT cell-dependent protective role. Notably, MAIT cells from patients with active TB show reduced IFN-γ production, likely by blocking both the γc and IL-2Rβ receptors and are regulated by PD-1 signaling [93,94]. Hence, impairment of MAIT cell functioning in combination with possible promotion of T cell exhaustion may be the strategies employed specifically by *Mtb* to evade recognition. Clearly, a more comprehensive understanding of MAIT cell activation and dysregulation in relation to TB infection is needed, and while the use of reMAIT to replenish MAIT cells holds promise as a future immunotherapy, its applicability against *Mtb* infection warrants more extensive investigation.
